# Supplementary material for: Are carbon emissions trading and green financial instruments synergistic? -Comprehensive quantitative research based on content analysis
Source: PLoS One. 2024 Mar 7;19(3):e0298601. doi: 10.1371/journal.pone.0298601 (PMC10919601; doi:10.1371/journal.pone.0298601)
Supplement: S1 Data — (ZIP) [file pone.0298601.s001.zip › Data/地级市Z_F工作报告.zip!/─■╧─/╬Γ╓╥╩╨/└┤╫╘╣1⁄2╓┌║┼í╛┬φ┐╦╩2╛▌═°í┐_2016.docx]

各位代表：

　　现在，我代表市人民政府，向大会报告政府工作，请各位代表与《吴忠市国民经济和社会发展第十三个五年规划纲要(草案)》一并审议，并请市政协委员和其他列席人员提出意见。

2015年工作及“十二五”回顾

　　2015年，面对复杂多变的发展环境和经济下行压力持续加大的严峻形势，在自治区党委、政府和市委的坚强领导下，全市上下以攻坚克难的勇气，真抓实干的作风，勇于担当的精神，接力“两场赛跑”，决胜“四仗一战”，统筹推进稳增长、促改革、调结构、惠民生等各项工作，保持了经济社会持续健康发展的良好势头。全年完成地区生产总值403.9亿元，增长7.5%；一般公共预算收入31.97亿元，下降5.7%；一般公共预算支出161.3亿元，增长11.3%；固定资产投资686.4亿元，增长14%；社会消费品零售总额95.3亿元，增长7.6%；城镇和农村常住居民人均可支配收入分别达21552.9元和9150.4元，增长8.6%和8.4%。

　　(一)精准发力，经济保持稳健运行。去年伊始，我市工业增速放缓，企业利润下降，奶产业出现波动。面对不利形势，我们科学研判，牢牢抓住政策扶持、金融服务、项目带动三个关键。积极落实自治区“工业18条”，兑现扶持资金2.2亿元，注资5000万元设立奶产业风险基金，举办葡萄酒品鉴推介会，盘活麒阳、宝迪等因资金链断裂停建的投资项目。与平安银行合作建立30亿元农业产业引导基金，22家企业在新三板等股权交易平台挂牌上市，嘉泽发电申报主板上市。新设立金融分支机构11家，新增小额贷款公司15家，成为全区唯一实现村镇银行县区全覆盖的地级市。争取项目1710个，落实批复资金127.9亿元，实施招商引资项目329个，到位资金558.2亿元。争取国家专项建设基金25.8亿元，居全区五市第二。优化投资结构，地方类产业投资占固定资产投资的94%，民间投资占比达到60%以上。

　　(二)培优扶强，转型升级步伐加快。工业转型步伐加快。伊利二期、安慕希酸奶项目建成投产，食品工业产值突破200亿元，增长20%以上。中国自动化产业园加快建设，杭萧产业园5家企业投入生产，装备制造业增速达50%以上。恒丰纺织规模不断扩大，恒和织布建成投产，集生态纺织、植物印染和服装加工为一体的全产业链正在形成。中民投盐池、同心光伏发电等新能源项目加快推进，风电光伏建成及在建规模达到698万千瓦，占全区“半壁江山”。全年新增规上企业50户，规上工业实现增加值152.2亿元，增长8.2%。万元GDP能耗下降17.7%，单位工业增加值能耗下降20.8%，降幅居全区首位。现代农业提质增效。粮食生产“十二连增”。新建千头以上生态牧场11个，奶牛存栏19.5万头，肉牛和滩羊饲养量分别达到60万头和620万只。瓜菜、有机枸杞、酿酒葡萄等优势特色产业规模不断扩大。利通区被农业部评为第三批国家现代农业示范区，红寺堡镇被评为中国葡萄酒第一镇，同心县易捷庄园成为全国集中连片最大的有机枸杞基地。培育销售过亿元龙头企业14家，扶持发展国家级专业合作社42家。全年实现农业总产值108.95亿元，增长4.7%。第三产业显现旺盛活力。探索“互联网+商贸”新业态，绿金在线等电商平台上线运行，电商交易额突破5亿元。盐池县、同心县被商务部列为电子商务进农村示范县。积极谋划发展健康产业，利通区健康产业园开工建设。大禹文化园等旅游项目对外开放，青铜峡市荣获2015最美中国“特色魅力、休闲度假”旅游目的地城市。利通区牛家坊等7个村被评为中国乡村旅游模范村。盐池县建成全区首个通用机场，红寺堡区成功承办2015全国青少年航空航天模型锦标赛。全年接待游客402.5万人次，实现旅游收入22亿元，分别增长21.9%和29.7%。

　　(三)提质增容，城乡建设内涵提升。实施城乡建设项目189个，完成投资158亿元，全市城镇化率达到55.3%。完成黄河过境段综合整治二期工程，建成乃光湖湿地公园。银西高铁、吴忠至中卫段城际铁路等重大项目开工建设。铺设供热、供排水管网47.5公里，新建续建城市道路95.9公里，综合改造老旧小区14个，完成老旧小区既有建筑节能改造110万平方米。新建美丽小城镇7个、美丽村庄37个，改造危房5000户，新修农村公路420公里。全市完成营造林32.4万亩，森林覆盖率达到15%，市区城市建成区绿地率、绿化覆盖率、人均公园绿地面积分别达到40%、41%和20平方米。扎实推进国家环保模范城市创建工作，拒引金属钠等污染项目13个。全市环境空气质量优良天数270天，优良率74%，位列全区沿黄城市首位。

　　(四)创新驱动，改革开放深入推进。我市被列为全国中小城市综合改革试点市，启动170项改革任务，完成148项。提前公布实施权力清单和责任清单，精简行政权力1188项，制定责任事项18512项。在全区率先推行“三证合一”，商事登记制度改革走在全国前列。农村土地承包经营权确权登记实现全覆盖。青铜峡市成立全区首家农民股份合作经营实体，利通区农村产权交易中心挂牌运行。在全区率先成立不动产登记局和不动产登记事务中心。吴忠仪表工业云平台被工信部列为全国首批工业应用示范平台。大坝发电等3家企业被列为国家级“两化融合”标杆示范企业。金融扶贫盐池模式、文化市场“执法+协会”管理模式在全国推广。香港晶澳等10家外资企业落户吴忠，台湾巨皇盐池体育馆及商业综合体项目成为海峡两岸(宁夏)经贸合作交流活动周首个落地项目。与北京西城区、天津武清区、上海青浦区、广西崇左市结为友好城市。开展友好城市回访活动，拓展了对外交流合作深度广度。全年外贸进出口总额1.2亿美元，增长21%。

　　(五)改善民生，百姓福祉持续增进。无论压力多大、财政多难，在保障改善民生上坚决不打折扣。全市财政投入民生领域资金达137.5亿元，占财政预算支出的85%。10件民生实事全面完成。整合投入各类资金10.3亿元，推动精准脱贫，大力推进龙头带动型等产业扶贫模式，创新推进光伏扶贫、乡村旅游扶贫，40个重点贫困村脱贫销号，减少贫困人口2.5万人。建成移民住房1395套，搬迁安置移民8576人，“十二五”生态移民任务全面完成。我市扶贫考核居五市第一，盐池县、青铜峡市分列山区、川区组第一。同心县扶贫办主任马希丰荣获2015中国消除贫困奖感动奖。改造棚户区1.8万套，回购商品房8000套，新建公共租赁住房1000套，一大批城乡困难群众喜迁新居。大力推进大众创业、万众创新，发放各类创业担保贷款7.4亿元，培育小企业784个、小老板1551人，城镇新增就业1.54万人，农村劳动力转移就业23.6万人，城镇登记失业率控制在4%以内，我市被确定为全国百城技能振兴专项活动城市。涌现出腾辉民族服饰、大学生电商创业平台等一批创业典型。城乡居民基本养老保险、医疗保险参保率分别达到90%和98%，城乡居民基础养老金、最低生活保障金每人每月分别增加15元和60元，大病医疗保险实现全覆盖。积极筹措2.89亿元，妥善解决利通区2209名被征地农民养老保险遗留问题。安排438万元对3097名特困群众进行临时救助，确保温暖过冬。建设居家养老服务站和农村幸福院98个。新建改建中小学校、幼儿园82所。高考一二本上线率28.26%，高于全区平均水平。创新发展职业教育，成立吴忠职教联盟。扎实推进国家卫生城市创建和县级公立医院综合改革，我市被确定为全国城市公立医院综合改革试点市。人口计生工作稳步推进。全国文明城市创建工作全面展开。成功获批第三批国家公共文化服务体系示范区。

　　(六)依法行政，社会大局和谐稳定。高度重视并自觉接受人大法律监督和政协民主监督。办理人大代表议案建议77件、政协委员提案175件，办复率100%。成立吴忠市决策咨询委员会，聘请政府顾问125名。创新开展“广场说法·一周一法”系列法制宣传活动，“六五”普法顺利通过自治区验收。加强行政监察和审计监督，举办“电视问政”13期，解决了一批群众反映强烈的突出问题。全区群众评议机关和干部作风活动五市排名第一。深化“平安吴忠”建设，严厉打击各类违法犯罪活动，八类刑事案件破案率等三项指标继续保持全区五市第一。新一轮禁毒人民战争成果丰硕。大力培育社会主义核心价值观，弘扬道德风尚正能量。市地税局荣获全国职工职业道德建设先进单位称号。蒯月琴等4人荣登中国好人榜，马跃霞等3人荣获最美宁夏人物，王金香家庭荣获全国最美家庭称号。成功创建为全国民族团结进步示范市，五个县(市、区)全部被命名为全国民族团结进步示范县。具有吴忠特色的“585”创建模式成为全国品牌，宗教人士争做“八大员”做法荣获中央统战部实践创新奖。积极推动军地、军民深度融合发展，以全区第一的好成绩顺利通过全国双拥模范城考评验收。安全生产形势总体稳定，各类事故起数同比下降22.16%。信访批次和人数分别下降9%和5%。应急管理、防震减灾、新闻出版、文史社科、妇女儿童、统计、档案、外事、气象、人防、老龄、慈善、残疾人等工作取得新成绩。

　　各位代表，回眸“十二五”时期，是我市发展极不平凡的五年。全市上下迎难而上、负重拼搏、勇于创新，破解了一道道难题，应对了一次次挑战，开创了各项事业发展新局面，“五个吴忠”焕发出蓬勃生机和旺盛活力。今日的吴忠，“玉在椟中求善价，钗于奁内待时飞”。

　　五年来，面对经济下行压力持续加大的发展环境，我们定向施策、精准发力，综合实力再上新台阶。全市地区生产总值从2010年的217亿元增加到403.9亿元，年均增长10.5%，比全区平均增速高0.6个百分点。一般公共预算收入从2010年的15.7亿元增加到31.97亿元，年均增长15.3%。固定资产投资五年累计达到2426.8亿元，是“十一五”的3.7倍，年均增长25.9%。社会消费品零售总额从2010年的52亿元增加到95.3亿元，年均增长12.9%。五年累计争取资金544亿元，建成了一批打基础、利长远的重大项目。

　　五年来，面对既要保增长又要调结构的双重压力，我们保持定力、多措并举，转型发展迈出坚实步伐。三次产业由2010年的175132调整到现在的13.256.230.6。轻重工业比由2010年的2278调整到2971，轻工业占比高于全区10.8个百分点。新增规上工业企业200家，工业增加值比“十一五”末翻了一番，“五优一新”工业占到70%以上。农业增加值比“十一五”末增长47.8%，特色农业占农业总产值90%以上。第三产业增加值比“十一五”末增长71.6%。“十二五”节能降耗目标任务提前完成。调结构、转方式取得突破性进展,转型发展走在了全区前列。

　　五年来，面对山川城乡发展差距仍然较大的现实，我们统筹兼顾、协调推进，城乡建设品质不断提升。五年累计实施城乡建设项目1057个。城市建成区面积由2010年的58平方公里扩展到125平方公里，新增公路里程2087公里。利青一体、银吴同城进程加快。红寺堡区、盐池县、同心县城市功能不断完善，辐射聚集能力持续增强。金积、叶盛、王团等一批中心集镇旧貌换新颜，累计建设美丽村庄92个。生态环境持续改善，森林覆盖率比“十一五”末提高3个百分点，空气质量优良天数连年位居全区沿黄城市首位。荣获中国人居范例奖，先后获得国家园林城市、全国绿化模范城市、全国建筑节能示范市等称号，成为全区唯一的全国生态文明建设试点市。

　　五年来，面对体制机制等方面的诸多瓶颈制约，我们大胆探索、重点突破，发展活力进一步释放。建成统一高效的政务服务平台。36家企业通过资本市场挂牌融资。农村产权、行政审批、金融扶贫等改革走在全区乃至全国前列。培育国家级高新技术企业3家、自治区级技术创新中心等研发平台30个。五年新增市场主体3.3万户，总量达到8.6万户，位居五市第二。成功举办三届“黄河金岸”国际马拉松赛。与国内外20多个城市建立友好合作关系。五年累计招商引资2315亿元，是“十一五”的4.6倍。

　　五年来，面对财政收支矛盾不断加剧的困难局面，我们坚持底线思维、优先保障民生，人民群众共享更多发展成果。每年公共财政支出80%以上用于民生事业。五年办理民生实事56件。城乡居民收入年均实现两位数增长，农村居民收入增幅连续六年高于城镇居民。累计投入61.2亿元，搬迁安置生态移民1.57万户6.8万人，减少贫困人口14.3万人，贫困发生率下降10.3个百分点。养老、医疗等五大保险全面建立，城乡低保实现应保尽保和提标扩面，10万被征地农民养老保险问题得到妥善解决，城乡居民基础养老金增长145%。城镇新增就业6万人，转移农村劳动力就业96万人，被评为全国创业先进城市。五年新建改建中小学186所、幼儿园47所，利通区、青铜峡市顺利通过国家义务教育均衡发展评估验收。实施市人民医院迁建、黄河文体中心等一批重大民生项目，村卫生室、农家书屋覆盖所有行政村。依法治市深入推进，成为全区首个全国法治城市创建先进单位。

　　五年来，面对千帆竞发、百舸争流的区域发展态势，我们加强协调指导、因地制宜，县域经济不断发展壮大。依据产业优势和资源禀赋，引导县区错位发展。利通区综合经济实力稳居全市前列，青铜峡市地区生产总值突破百亿元大关，红寺堡区、同心县主要经济指标全部实现翻番，盐池县综合实力领跑山区县。

　　五年的实践使我们深深体会到：只有坚持发展不动摇，始终坚定发展信心，保持战略定力，不松劲、不懈怠、不折腾，才能走出一条质量更高、效益更好、结构更优、优势充分发挥的发展新路。只有以开阔的视野、全局的观念、改革的思维、创新的精神，敢于破除体制机制障碍，敢于解决深层次矛盾，才能为经济社会持续健康发展提供新动力、开辟新空间。只有始终坚持正确方向，汇聚各方力量，形成以上率下、上下齐心的工作状态，风清气正、干事创业的政治生态，才能凝聚推动发展的强大合力。只有以百姓之心为心，多办顺民意、暖民心、释民怨的实事好事，苦干实干，用干部的“辛苦指数”换取群众的“幸福指数”、企业的“效益指数”，才能赢得百姓的认同、企业的信赖和各界的支持。

　　各位代表!去年一年及“十二五”时期的成绩来之不易。这是自治区党委、政府正确领导的结果，是市委总揽全局、科学谋划的结果，是人大、政协有力监督、大力支持的结果，是全市上下精诚团结、奋力拼搏的结果，也是一任接着一任干，持续奋斗、久久为功的结果。在此，我代表市人民政府，向奋战在各条战线的广大干部群众，向人大代表、政协委员、各民主党派、工商联，向离退休老同志和社会各界人士，向驻吴单位、驻吴部队和武警官兵致以崇高的敬意!向关心支持吴忠发展的国家部委、自治区厅局、友好城市、政府顾问和外商、港商、台商表示衷心的感谢!　　在看到成绩的同时，我们也清醒地认识到，我市发展中还存在着一些深层次问题。一是发展的困难依然较多。总量小、底子薄、基础弱，发展不足仍是最大市情。资源环境约束不断加大，农业发展“水瓶颈”日益凸显，山川发展不平衡出现新的趋势。财政收支矛盾加剧，还债压力较大。二是发展的质量依然不高。产业结构不尽合理，工业支撑作用不强，税收就业贡献不多。农业龙头企业示范带动作用不够，品牌影响力不大。传统服务业占比偏大，现代服务业相对滞后。科技创新能力不强，创新人才短缺。三是发展的任务依然艰巨。保障民生刚性支出增大，教育、医疗、文化等公共服务水平与群众期望还有差距。扶贫攻坚任务艰巨，脱贫摘帽压力较大。政府职能转变需要不断深化，作风建设还需持久用力，等等。对这些问题，我们必须高度重视，认真研究，切实解决。

“十三五”规划蓝图和主要任务

　　“十三五”时期，是全面建成小康社会的决胜期，也是深化改革、转型发展的攻坚期，更是破解难题、补齐短板的关键期，是我们必须紧紧抓住并且可以大有作为的重要战略机遇期。

　　根据市委“十三五”规划建议，今后五年我市经济社会发展的总体要求是：高举中国特色社会主义伟大旗帜，全面贯彻党的十八大、十八届三中、四中、五中全会和习近平总书记系列重要讲话，以及自治区党委十一届七次全会、自治区十一届人大五次会议和市委四届九次全会精神，协调推进“四个全面”战略布局，坚持发展第一要务，以创新、协调、绿色、开放、共享发展理念为引领，以提高发展质量和效益为中心，以转型发展、脱贫攻坚为主线，以改革开放为动力，加快形成引领经济发展新常态的体制机制和发展方式，统筹推进经济建设、政治建设、文化建设、社会建设和生态文明建设，开创开放、富裕、和谐、美丽、智慧吴忠建设新局面，确保如期全面建成小康社会，谱写伟大复兴中国梦的吴忠新篇章。

　　主要预期目标是：地区生产总值年均增长8%左右，城镇和农村常住居民人均可支配收入年均分别增长9%和10%。到2020年，经济总量、城乡居民收入在2010年基础上翻一番半。固定资产投资年均增长12%以上。一般公共预算收入与经济增长同步。农村贫困人口提前两年全部脱贫，贫困县全部摘帽。完成自治区下达的节能减排任务。

　　各位代表，这样一个预期目标，是我们正视发展差距、回应群众期盼、着眼同步全面小康，在科学研判和理性分析的基础上确立的，也是我们未来五年矢志不渝的奋斗方向。实现上述目标，必须坚持把转型发展作为首要任务，必须坚持把融合发展作为基本路径，必须坚持把补齐短板作为重中之重，必须坚持把依法行政作为根本保证。

　　今后五年，我们要在以下八个方面谋求新突破、取得新进展：

　　产业结构更加合理。以“一特三高”为引领，打响农业“绿色、有机、富硒、健康”品牌，推动草畜、优质粮食、酿酒葡萄、有机枸杞、中药材、瓜菜、家禽、休闲农业八大优势特色产业精品化、高端化、品牌化。打造“塞上硒都”、建设孙家滩生态农业“有机谷”。培育新型经营主体和特色产业基地，延长产业链，提高附加值。农业总产值突破140亿元。加快实施中国制造2025宁夏行动纲要，促进工业化和信息化深度融合。主攻装备制造、能源化工、新能源产业提升，加快构建油气电力、生态纺织、新能源等10个“百亿元”板块，聚力打造食品产业、健康产业2个“千亿元”产业，培育产值过50亿元企业8家左右，10亿元20家左右。全市工业总产值突破千亿元大关。重点推进旅游与文化、体育、度假等融合互动，加快发展电子商务、金融保险、养老服务等新业态。现代服务业占第三产业比重达到60%以上，第三产业增加值占GDP比重达到30%。

　　基础设施更加完善。实施一批重大公共设施和基础设施项目，推进银西高铁、吴忠至中卫段城际铁路、银昆高速、银百高速等过境铁路和公路建设，高标准建设吴忠客运站和货运站，加快吴忠至宁东、利通区至盐池县、青铜峡市至银川等快速通道建设，打通慈善大道、罗山大道向南延伸段，打造区域重要交通节点，形成连接北京、兰州、西安等大中型城市“半日交通圈”。加快黄河吴忠段水运项目建设，着力打造集水运、观光、娱乐、生态为一体的黄金旅游水道。积极推进红寺堡通用机场及配套设施建设，规划建设吴忠支线机场。全面完成苦水河治理、扁担沟扬水灌区续建配套等水利工程。彻底解决农村人口饮水安全问题。实施中部干旱带中小型水库等工程建设。完善城市防洪排涝体系，建设“海绵城市”。黄河重点河段防洪标准达到50年一遇。

　　城乡发展更加协调。着力打造一个城市核心区、三个副中心城区、若干重点镇和新型社区组成的新型城镇化体系，走符合吴忠定位和实际的内涵式、集约化发展之路。深入推进户籍制度改革，全面实行居住证制度，常住人口城镇化率达到60%。加快利青一体发展，综合改造老旧小区，基本完成现有城镇棚户区、城中村和农村危房改造，逐步消除交通拥堵、环境污染、停车难等“城市病”。加快盐同红大县城和美丽乡村建设，集中建设一批重点特色镇，引导人口、产业集聚发展，全市所有乡镇和90%的规划村庄达到美丽乡村建设标准。以“智慧吴忠”为载体，建成公共基础数据库和信息平台，逐步实现城乡信息服务电子化应用。

　　生态环境更加优美。大力实施生态立市战略。建立生态文明考核评价、生态环境损害责任追究等制度，切实做到归属清晰、权责明确、监管有效。健全科学合理的生态保护和补偿机制，持续加大大气、水、土壤污染防治，继续实施禁牧封育、天然林保护、移民迁出区生态修复等工程，推进“三廊两网”建设，构筑绿色安全屏障。统筹做好节水、蓄水、调水文章，做到以水定产、以水定地、以水定城，提高水资源利用效益，建设节水型社会。整合黄河水、地表水等水资源，连通环城水系。健全资源利用回收体系，提高资源利用效率，争创国家循环经济示范城市。全市完成营造林150万亩，森林覆盖率达到17.5%。环境空气质量优良天数比例达到80%，城市PM10浓度比“十二五”末下降20%。建成国家生态文明建设示范市和国家环保模范城市。

　　改革开放更富活力。全面取消非行政许可审批事项，实现全市联网审批。构建多元金融服务体系，提高直接融资占比，力争5家左右企业在主板上市，努力建成宁夏区域性金融服务和结算中心。稳妥推进国有企业发展混合所有制经济，发挥投融资平台作用，提高国有资本运行效率。全力打造丝绸之路经济带“黄金节点”。支持吴忠企业在国内外设立特色产品展示展销中心，提升知名度和影响力。创新发展慈善事业，吸引更多中外慈善人士来吴投资兴业、奉献爱心。

　　创新引领更加深入。深入实施创新驱动战略。完善政府引导、企业为主体的科技投入稳定增长机制，全社会研究与试验发展经费占GDP比重达到2.1%。强化企业创新主体地位和主导作用，建立科技创新后补助机制和科技成果转化机制，支持企业、园区与区内外高等院校、科研机构组建博士工作站和技术研发中心，建设一批技术中心、技术创新实验室，培育一批国家级高新技术企业、自治区级科技创新企业，促进科技成果资本化、产业化，提升科技创新对产业转型升级和新业态培育发展的支撑能力。全面实施大众创业、万众创新行动，搭建众创平台，引进培育紧缺人才、创新人才，发挥专家智库作用，推进人才与产业融合发展。

　　人民生活更加幸福。牢固树立共享发展理念，持续推进民生改善。实施精准扶贫、精准脱贫，推广产业、金融、电商、光伏、乡村旅游、教育等扶贫模式。盐池县2017年摘帽，红寺堡区、同心县2018年摘帽，3年实现15.06万人脱贫、168个贫困村销号。决不让一个贫困地区、一名贫困群众在小康路上落伍掉队。建立更加公平可持续的社会保障体系，推动社会保险提标扩面，实现参保人群全覆盖。全面落实城乡居民大病医疗保险和大额医疗救助制度。完善社会救助体系，探索养老服务模式，实现残疾人医疗康复服务全覆盖。坚持教育优先发展，创新发展各类教育，让每一个孩子都能平等接受教育。推行医药分开、分级诊疗，实行医疗、医保、医药“三医联动”。加快发展文化体育事业，形成“二十分钟”公共文化服务圈。

　　社会大局更加稳定。深入推进依法治市。落实重大决策社会稳定风险评估制度，健全依法维权和矛盾纠纷排查化解机制，有效预防和化解矛盾纠纷。加强社会治安综合治理，加快社会治安防控体系建设，深入推进禁毒人民战争，各类刑事案件和治安案件发案率持续下降，不断提升人民群众安全感和满意度。严格落实安全生产一票否决和责任追究制度，加强事故应急救援保障体系建设。巩固提升民族团结进步创建成果，依法加强宗教事务管理，把民族团结、宗教和顺的名片打造得更加靓丽。加强精神文明建设，用“中国梦”、社会主义核心价值观凝聚共识、汇聚力量。深入推进全国文明城市创建工作，提升社会文明程度，展示城市良好形象。

2016年预期目标和主要工作

　　2016年是开局年，也是攻坚年，做好今年工作意义重大。

　　今年的主要预期目标和约束性指标是：地区生产总值增长8%左右，固定资产投资增长12%以上，一般公共预算收入增长7%以上，社会消费品零售总额增长8%以上，城镇和农村常住居民人均可支配收入分别增长9%和10%，城镇登记失业率控制在4%以内，居民消费价格涨幅控制在3%以内，全面完成自治区下达的节能减排任务。

　　重点抓好十个方面的工作：

　　(一)坚定不移抓项目、扩投资，切实增强发展后劲。谋划实施一批大项目、好项目，形成有效投资，全力拉动经济平稳较快增长。扎实推进项目建设。重点实施中国自动化产业园、杭萧汽车零部件制造产业园等产业项目，热电联产供热管网、城市东部地下综合管廊等基础设施建设项目，教育医疗文化、保障性住房等民生项目。准确把握国家、自治区项目资金投向，加强对接，确保上争资金130亿元以上。力推PPP模式，撬动社会融资，形成多元投资格局。加大项目引进力度。瞄准产业链高端，围绕新能源、装备制造、食品工业等优势特色产业，主动对接500强企业，争取引进10亿元项目10个以上，500强企业2家以上，5000万美元外资项目2个。招商引资实际到位资金突破570亿元。做好项目服务保障。严格落实重点项目包抓责任制，已批复或签约项目，抓紧做好项目选址、环评、征地等前期工作，及时开辟绿色通道，确保项目早日开工。已开工续建项目，加快进度，力争早日投产达效。创造良好施工环境，确保银西高铁、城际铁路、京藏高速改扩建等重大项目顺利推进。

　　(二)持之以恒调结构、转方式，加快工业转型升级。围绕优势特色，扩总量、调结构、增后劲，力争规上工业实现增加值167亿元，增长9%。做大工业总量扩实力。实施投资5000万元以上重点工业项目110个，新增规上企业30家。重点推动10个产值或销售收入过百亿元产业板块集群化发展，力争建成煤炭电力、油气化工、新材料3个产值过百亿元产业板块。整合改造工业园区水电路等配套设施，打造低成本工业园区。做强优势产业增效益。启动实施恒丰高档出口缝纫线、民族服饰等项目，拓展延伸上下游产业链，轻重工业比达到3070。确保华冠数控装备等项目建成投产，装备制造业产值保持50%以上增速。力争我市被列为中国制造2025试点城市。加快中民投等5个光伏产业园项目建设，力争新能源建成装机总容量达到500万千瓦。推动健康产业促转型。坚持项目引导，在实体产业培育方面取得实质性突破。加快推进利通区健康产业园、中医药研创基地、功能性产品研发中心、同心中医药健康体验馆、盐池甘草加工、联合国营养强化面粉试点等项目建设，打造区域性健康产业中心，力争健康产业增加值占GDP10%以上。改造传统产业添活力。大力支持企业加快技术改造，推进战略重组、行业整合，全力支持金牛化肥、金昱元化工、普华冶金等传统骨干企业加快转型升级。帮助吴忠卷烟厂提升产品档次，提高附加值。引导青铝等企业通过战略合作，调整产品结构，实现可持续发展。

　　(三)坚持不懈扩规模、做精品，推动农业提质增效。以农业增效、农民增收为核心，推动农业融合发展、转型升级，力争农业增加值增长5%以上。以产业化为重点，推动农业扩规上档。全市粮食生产稳定在320万亩、90万吨，新增奶牛1.5万头、肉牛5万头、滩羊20万只，瓜菜面积达到55.4万亩，有机水稻、有机枸杞、酿酒葡萄、中药材种植面积分别达到20万亩、10万亩、21.3万亩和54.3万亩。加快青铜峡鸽子山、红寺堡罗山葡萄产业示范区建设，加强与拉美、法国等国家地区技术、品牌合作，新建酒庄16个。积极发展生态型休闲观光旅游业，促进农业与其他产业融合发展。争创自治区级休闲农业示范县1个。以市场化为核心，培育农业产业品牌。支持正鑫源、易捷、润德等龙头企业走精深加工路子，打造高端品牌。大力推动富硒农畜产品研发基地建设，加快培育一批富硒企业和产品，富硒农产品种植面积达到44万亩。打响“同心有机枸杞”“孙家滩瓜菜”“青铜峡富硒大米”“盐池滩羊”等品牌。培育市级以上重点龙头企业10家，新型经营主体40家。加快在北京、天津、上海等11个城市设立优质特色农产品展示展销窗口。以科技化为途径，夯实农业发展基础。加大农业科技投入力度，发挥农业科技专家智库支撑和吴忠国家农业科技园区示范引领作用。因地制宜开展农田水利基本建设，探索试点沟渠日常化管理，适时适度退耕休耕轮耕，大力发展节水农业。完善追溯监管体系建设，加强“三品一标”认证，实现农产品监测全覆盖。

　　(四)千方百计挖潜力、提层次，释放三产后发优势。推动商贸物流、文化旅游、健康产业、养老服务等业态融合发展，促进服务业发展提速、比重提高、结构优化，力争第三产业增加值增长6.5%。拓展延伸现代服务业。编制电商发展规划，实施“互联网+”行动，推动“智慧吴忠”和信息化建设，争创全国电子商务示范市。放宽准入条件，重点开拓体育健身、健康与营养保健等领域，加快推进路捷千人养老公寓等项目建设。探索医养结合模式，培育中医中药、养生保健等新兴业态。持续推进服务外包、家政托幼、设计研发、文化创意等业态发展。发挥通用机场作用，发展通航产业和临空经济。改造提升传统服务业。加快现代物流体系建设，发展综合性物流中心6个，培育一批辐射全市、融入全区的大型专业市场，促进产业与物流紧密链接。深入开展特色餐饮业品牌提升活动，统一标识标牌，鼓励支持餐饮“走出去”发展连锁经营。融合推进文化旅游业。深化与台湾旅游企业、宁夏旅游集团等旅游机构合作，主动融入宁夏全域旅游，打造精品线路和精品景区，构建旅游经济共同体。大力发展参与式、体验式等新型旅游业态。鼓励文化创意、演艺与旅游资源整合，开发具有地域特色和民族风情的旅游演艺精品和旅游商品。力争全年接待游客和旅游收入分别增长15%和17%。

　　(五)锲而不舍抓统筹、增内涵，加快新型城镇化建设。坚持以人为中心的城市发展理念，实施城乡建设项目120个，投资130亿元，全市城镇化率达到56.3%。优化城乡规划空间。高起点编修新一轮城市总体规划、专项规划和重点区域详控规划，推动“三规合一”“多规融合”。加强对城市空间立体性、平面协调性、风貌整体性、文脉延续性等方面的规划管控，留住城市特有的地域环境、文化特色和建筑风格。打造宜居宜业城市。启动银西高铁枢纽站东片区道路、水系、绿化等配套设施建设，着力打造功能齐全、环境优美、风情独具的吴忠新门户、产业新平台、宜居新社区。实施城市绿道、供水水质提标改造等建设项目。新建续建城市道路110公里，铺设供排水管网66公里，综合改造37个老旧小区及散居楼，实施既有建筑节能改造120万平方米。坚持房地产市场导向，继续推行存量房回购政策，有效化解房地产库存。启动建设特色街区，提升城市魅力，保存乡愁记忆，注重群众的衣食住行、安居乐业，增强百姓的认同感和归属感。推动城乡融合发展。加快大县城建设，重点实施盐池县环城公园绿化亮化、同心县汽车客运站、红寺堡区第二污水处理厂等项目，进一步提升集聚承载能力。围绕特色产业和地域文化，打造旅游小镇、枸杞小镇、葡萄小镇等特色小城镇，吸引周边群众向中心镇聚集。高标准建设美丽小城镇5个、美丽乡村20个，改造农村危房3000户，新修农村公路400公里。提升城乡管理水平。大力实施城市净化、美化、绿化、亮化工程，启动垃圾分类试点，合理新增停车泊位，配套完善环卫设施，城市环卫机械化作业率达到80%。建设数字化管理平台，打造智慧城市。加强小区物业管理，推动物业标准化，提升居民满意度。健全城乡环境综合整治长效机制，继续开展生态文明示范乡镇和生态村创建活动，切实改善农村环境。充分发挥协同推进作用，实现城乡共治共管、共建共享，让群众生活得更方便、更舒心、更温馨。

　　(六)群策群力筑屏障、靓家园，着力打造美丽吴忠。坚守生态红线，加大生态治理和环境保护力度，让吴忠的天更蓝、地更绿、水更清、空气更清新。着力改善生态环境。加快推进国家生态文明建设示范市创建工作，健全全民义务植树、生态移民迁出区生态修复、公益林保护管理三项机制，统筹实施退耕还林、防沙治沙等林业工程，重点抓好绿化提质增景改造、主干道路绿化美化等工程。全市营造林21万亩，封山育林7万亩，生态移民迁出区修复面积5.69万亩，森林覆盖率达到15.5%，创造更多的绿色财富和生态资源。突出抓好环境治理。深入实施环境保护三项行动计划，强化负面清单管理，坚决不要带毒发臭的GDP，淘汰落后产能50万吨。加快推进环保产业园建设。狠抓农业面源污染治理。加快清水沟入河口生态湿地建设，加大南干沟重金属污染治理，建设工业园区污水处理设施。有效治理大气环境污染，确保环境空气质量全区领先。完善生态保护机制。严格落实环保准入制度，制定环保责任规定和督查考核办法，开展环境保护审计。完善资源有偿使用和生态补偿机制，大幅提高违法成本。积极开展节能量、碳排放权和排污权交易试点。编制建设项目环境影响评价分类管理目录，全力推动政府环保职能履行到位。

　　(七)凝心聚力促创新、推改革，激发发展动力活力。紧扣关键，持续用力，破除障碍，把改革开放的红利转化为发展新动能、新优势。继续深化行政审批改革。完成“政务云”行政审批和公共便民服务系统建设，大力推行网上审批、“一门受理、内部流转、多证联办”审批服务模式。巩固扩大“三证合一”“一照通”商事登记制度改革成果，进一步激发市场主体活力。稳步推进不动产登记制度、户籍制度等重点领域改革。纵深推进财税金融改革。推进“全口径”预算体系建设，做好存量债务置换，切实提高财政资金使用效益。加强税收征管，完成税制改革任务。深化与宁夏股权托管交易中心战略合作，拓宽融资渠道，力促嘉泽发电主板上市，20家企业在新三板及区域股权托管交易中心挂牌。开展金积工业园区首批中小企业集合债试点发行工作。持续推进国资国企改革。加快市属国有企业改革，发挥城投公司、国资运营公司、水投公司等投融资平台作用。组建城市公交、农业扶贫、文化旅游、中小企业创业等投资公司，盘活国有资产。建立健全奶产业、酿酒葡萄等产业发展基金和风险担保基金。以开源担保公司为试点，探索混合所有制改革，扩大担保规模。稳步推进农业农村改革。全面完成农村土地确权，推进宅基地和农宅“两证合一”，试点开展农民闲置宅基地置换、转让、公开竞拍和有偿退出，盘活闲置土地资产。有序推进农村集体资产股份制改革，量化集体净资产，促进村集体经济发展壮大。大力推进集体林权流转、抵押质押贷款和森林保险改革。加快推进水权交易试点，实行水资源公开有偿交易。巩固扩大开放合作成果。实施开放吴忠建设十大行动。精心筹办同心、盐池80周年纪念活动，贺兰山东麓葡萄酒文化节等节展活动，扩大对外影响。建立丝路经济带联合会、吴忠台湾商会等对外开放合作平台。加强与中俄蒙友好事业发展有限公司合作，促进外贸合作和产业对接。积极促成浙江数贝有限公司与国电吴忠热电有限公司合作，共建云金融数据中心。协调银川海关、银川综合保税区开辟“绿色通道”，支持企业开拓国际市场，力争进出口总额达到1.5亿美元，增长20%。全面实施创新驱动战略。设立科技创新发展基金，探索建立科技成果转化经纪人制度，激发创新活力，催生成果转化。深化与中国营养学会、清华大学等科研院所合作，争取设立院士工作站和博士服务点，建设科技创新平台7个。力争金积工业园区进入自治区高新技术园区行列，上海中海龙高新技术研究院吴忠分院挂牌运行。加大“招才引智”力度，建立服务对接责任落实机制，切实发挥决策咨询委员会和政府顾问智库作用，为经济社会发展提供智力支撑。

　　(八)全心全意惠民生、促公平，提升群众幸福指数。优先安排民生项目，切实做到保基本、兜底线、促公平、可持续，让人民群众生活更加殷实富足、更加幸福安康。打赢脱贫攻坚战役精准脱贫。实施脱贫攻坚八项行动计划，出台脱贫攻坚责任清单，建立精准脱贫全面量化管理和绩效考核体系。启动实施新一轮生态移民工程，妥善解决生态移民遗留问题。落实扶贫和农村低保标准“两线合一”，将完全或部分丧失劳动能力的贫困人口全部纳入低保。扎实做好定点帮扶、社会帮扶。确保脱贫销号重点贫困村65个，减少贫困人口5万人。提高社会保障水平兜牢底线。落实创业职业培训、贷款贴息、创业孵化等政策措施，力争新登记市场主体增长20%以上，实现城镇新增就业1.4万人，转移农村劳动力20万人以上。加快“五险合一”经办体制改革，稳步扩大医疗、失业、工伤和生育等社会保险覆盖面，提高非公有制企业、灵活就业人员、农民工参保率。完善社会救助体系，落实临时救济政策，让每一个困难群众都能得到及时救助。加强保障性安居工程建设，新建公共租赁住房1000套，实施棚户区改造3000户，让更多困难家庭安居乐业。创新发展慈善事业，年内建成市残疾人康复中心，让残疾人群体得到更多关爱，生活更有信心。协调发展社会事业促进公平。全力办好承诺为民办的10件实事。新建改建幼儿园17所、中小学30所，切实改善农村贫困地区薄弱学校办学条件。稳步提高学前三年幼儿入园率。加大城乡校长教师交流轮岗力度，扩大优质教育资源覆盖面。落实免除农村贫困家庭普通高中和中等职业教育学杂费政策，巩固普及高中阶段教育，推动职业教育内涵式、特色化发展。深化公立医院综合改革，支持市级医院与基层医疗机构建立医疗联合体，降低就医成本。开展中小学生口腔健康检查。扎实推进国家卫生城市创建工作，确保通过国家评估验收。改革完善计划生育服务管理，认真落实全面两孩政策。全面实施第三批国家公共文化服务体系示范区创建工作。鼓励全民健身，公共体育场馆免费或低收费向社会全面开放。高标准承办第五届“黄河金岸”国际马拉松赛，及早筹备自治区第十五届运动会。

　　(九)尽心竭力促团结、保稳定，维护社会和谐局面。突出基层基础建设和体制机制创新，注重管理服务并重和文明水平提升，激发社会活力，维护社会稳定，促进社会和谐。创新社会管理保平安。启动“七五”普法工作，落实人民调解、行政调解和司法调解联动机制，建立吴忠市法律援助窗口，打造“城市半小时、农村一小时”法律援助服务圈。构建农民工工资支付诚信体系，切实保障农民工合法权益。开展电动车综合管理整治。加大社会治安综合治理力度，严厉打击各类违法犯罪活动。努力建设禁毒工作示范市，坚决打赢新一轮禁毒人民战争，绝不让毒品犯罪抬头，毒品危害蔓延。强化安全生产红线意识，严格落实党政同责、一岗双责，加强食品药品、特种设备安全监管，坚决杜绝重特大安全事故。加强民族宗教工作促和顺。深入实施民族团结“八大工程”，巩固扩大民族团结进步示范市创建成果。依法加强宗教事务管理，及时化解宗教领域矛盾纠纷，依法打击非法宗教活动，把民族和睦、宗教和顺、社会和谐的大好局面巩固好、发展好。提升文明程度聚能量。深入推进全国文明城市创建工作，深化文明单位、文明行业、文明社区、文明村镇创建活动，提高社会文明水平。进一步加强国防动员，持续推进双拥共建，密切军民军政关系，为国防建设和部队官兵多办实事好事，促进军地融合发展。加强防震减灾、文史社科、外事侨务、档案、气象等工作。支持工会、共青团、妇联等群团组织创新开展工作。

　　(十)久久为功转作风、优环境，建设人民满意政府。适应新常态，谋求新发展，完成新任务，对政府自身建设提出了更新更高更严要求，政府部门和全体工作人员必须主动适应，自觉践行，以“三严三实”要求加强自身建设，提振精气神、担当新使命。做到科学理政。更加注重学习，锤炼干事本领，增强统筹能力，提升在新常态下推进工作的能力水平。广泛听取社会各界意见建议，博采众智，集思广益，安排部署工作讲科学、重理性、循规律、不蛮干、不任性，使之更加符合客观实际，更加体现人民意志。注重发现苗头性、倾向性问题和趋势性变化，未雨绸缪，提前应对，牢牢把握发展主动权。突出依法行政。一丝不苟地落实权力清单和责任清单，切实做到“法无授权不可为、法定职责必须为”。健全完善重大行政决策公众参与、专家论证、风险评估等制度。自觉接受人大法律监督和政协民主监督，高度重视司法监督、舆论监督和公众监督。始终用法治引领改革发展、促进社会公正。强化服务简政。推进政务公开，建立新闻发布制度。着力构建便捷高效服务环境，努力打造投资“洼地”、服务“高地”、发展“福地”，既让企业家放手创新，更让企业家放心发展，让创业者真正感到在吴忠发展顺风顺水、大有作为。坚持高效施政。干字当头、实字为要。定下的事情，抓紧实施，及早完成；部署的工作，善始善终，善作善成。坚持以目标倒逼进度、以督查强化落实，确保各项工作高效运行。坚决整治不作为乱作为，坚决克服懒政怠政，坚决惩处失职渎职。以我们的“勤奋指数”换取吴忠的“发展指数”。抓好廉洁从政。践行“三严三实”，反“四风”，刹歪风，优化政风行风，严格执行廉洁自律各项规定。强化行政监察和审计监督，扎紧织牢制度围栏，从严查处违纪违法案件，打掉寻租空间，铲除腐败土壤，营造风清气正的干事创业环境，推进作风建设常态化。严控“三公”经费和一般性支出，用政府的紧日子换来群众的好日子。

　　各位代表，梦想承载希望，实干开创未来。天时人事日相催，冬至阳生春又来。让我们高举中国特色社会主义伟大旗帜，在自治区党委、政府和市委的坚强领导下，团结全市人民，同心协力、锐意进取、务实苦干，为建设开放、富裕、和谐、美丽、智慧吴忠，建成全面小康社会而努力奋斗!
